# Carrageenan@Graphene Oxide Nanocomposites for the Adsorption of Trivalent Arsenic, Hexavalent Chromium, and Fluoride Ions from Aqueous Solutions: Conceptualization Modeling of Adsorptive Interfacial Interactions, Kinetics, and Swelling

**DOI:** 10.1021/acs.langmuir.5c01927

**Published:** 2025-06-23

**Authors:** Anastasia D. Meretoudi, Athanasia K. Tolkou, Ramonna I. Kosheleva, Maria Xanthopoulou, Nikolaos M. Tzollas, Margaritis Kostoglou, Ioannis A. Katsoyiannis, George Z. Kyzas

**Affiliations:** † Hephaestus Laboratory, School of Chemistry, Faculty of Sciences, 37791 Democritus University of Thrace, GR-65404 Kavala, Greece; ‡ Laboratory of Chemical and Environmental Technology, Department of Chemistry, 37782 Aristotle University of Thessaloniki, GR-54124 Thessaloniki, Greece

## Abstract

This research aims to develop low-toxicity, cost-effective, and reusable biobased materials by combining natural biopolymers with graphene oxide. Therefore, chitosan/kappa carrageenan (CS/kCar) and chitosan/kappa carrageenan/graphene oxide (CS/kCar@GO) nanocomposites were synthesized and applied for the effective removal of As­(III), Cr­(VI), and F^–^ from aqueous solutions. The results showed that the maximum removal for As­(III) (79%), Cr­(VI) (99%), and F^–^ (36%) was achieved at pH 5.0, 7.0, and 3.0, respectively. The data were best fitted by pseudo-second-order (PSO) kinetics and the Langmuir isotherm model, indicating adsorption capacities of 2.44 for As­(III), 2.82 for Cr­(VI), and 32.63 mg/g for F^–^ from the optimal CS/kCar@GO composite. According to the thermodynamic analysis, the adsorption of heavy metals and fluoride ions on the CS/kCar@GO nanocomposite was endothermic and spontaneous in all cases. Reuse of CS/kCar@GO for up to 10 cycles after regeneration demonstrated the effectiveness of this adsorbent.

## Graphical abstract

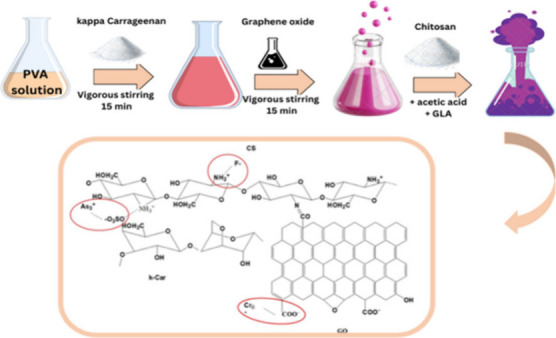

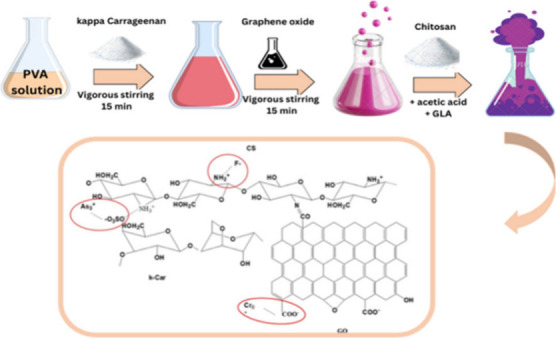

## Introduction

The frequent use of heavy metals and fluoride ions in numerous fields, including medicine, agriculture, and industry, has led to their uncontrolled release into the environment.^1^ Heavy metals are known for their toxicological effects, which include neurological damage, human organ harm, and cancer.^2^ In addition, arsenic is a major environmental contaminant due to its natural occurrence, toxicity, and carcinogenic potential.^3^ As­(V) (arsenate) and As­(III) (arsenite) are the two oxidation states of arsenic that are known to be highly toxic.^4^ More than 200 million people are exposed to high levels of arsenic in potable water,^3^ and the EPA (U.S. Environmental Protection Agency) and WHO (World Health Organization) have set the arsenic standard permissible limit for drinking water at 0.01 mg/L.^5^

Additionally, even if Cr­(III) is essential for nutrition and metabolic functions in the human body,^6^ Cr­(VI) is the hazardous oxidation state that causes several health effects in humans.^7^ Cr­(VI) is a significant groundwater pollutant, as it migrates more easily than other heavy metals.^8^ Given its teratogenic and carcinogenic properties, Cr­(VI) is considered one of the most concerning contaminants in groundwater^9^ and WHO set a tolerance limit of 0.05 mg/L in potable water.^10^ On the other hand, fluoride pollution, affecting more than 200 million people globally, also poses serious health risks.^11^ At low concentrations (i.e., <1.2 mg/L), fluoride ions are effective in preventing tooth decay, while at high concentrations (i.e., >1.5 mg/L), fluoride ions can lead to dental and skeletal fluorosis, endocrine disruptions, attention deficit hyperactivity disorder (ADHD), and neurotoxicity.^12^ To ensure global health, the WHO has set the maximum permissible limit for fluoride ions (F^–^) at 1.5 mg/L.^5^

Various treatment methods such as AOPs, adsorption,^13^ chemical precipitation, flocculation, flotation, and electrochemical methods^14^ have been developed to mitigate the toxicity of heavy metals^15^ and fluoride ions.^16,17^ However, identifying an effective and cost-efficient method remains a significant challenge. Among these methods, adsorption^18^ is an economic and low-impact technique for treating wastewater, especially at lower concentrations.^19,20^

Carrageenan is a hydrophilic anionic polysaccharide, consisting of a linear structure consisting of d-galactose and 3,6-dehydro-d-galactose residues.^21^ In addition, chitosan contains amino and hydroxy groups, which makes it suitable for surface modification, thus enhancing adsorption efficiency.^22^ Chitosan is positively charged, while carrageenan is negatively charged.^23^ In aqueous systems, polycations and polyanions typically form aggregates or polyion complexes due to strong electrostatic interactions, rather than creating a homogeneous solution. Furthermore, graphene oxide (GO) is a promising adsorbent for arsenic removal;^24^ however, it faces challenges like self-agglomeration and difficulties in separating nanoparticles during post-treatment.^3^ If polymer molecules could be chemically bonded or interact within GO’s layered structure, these limitations might be reduced, significantly improving the adsorption performance of the material. GO sheets can provide strong mechanical stability and durability by being attached firmly to chitosan, forming flexible configurations.

This work focuses on the development of low-toxicity and reusable biobased materials by the combination of natural biopolymers with graphene oxide, i.e., synthesized CS/kCar@GO. These novel composite materials may be economically and environmentally sustainable materials, appropriate for long-term use in pollution remediation, as the combination of chitosan (CS) and kappa carrageenan (kCar) results in the formation of a stable system.^25^ Moreover, poly­(vinyl alcohol) (PVA) is a cheap, nontoxic polymer and suitable for reactions with various functional groups.^26^ Its long polymer chains favor the formation of high-density hydrogen bonds with chitosan, strengthening the aerogel’s mechanical properties through robust interpolymer bonding.^27^ When fully hydrolyzed and blended with chitosan, PVA increases the number of hydroxy groups on the chitosan structure.^28^ In addition, addition of PVA to polysaccharide mixtures enhances the mechanical properties of the adsorbent material, as reported by Lui et al.^29^ This is because PVA is a synthetic polymer that can act as a cross-linking agent and matrix component, contributing to the overall stability and strength of the adsorbent.^30^ As a consequence, the addition of PVA to adsorbent materials indirectly affects the adsorption process by increasing the life span of the composite.

In this work, PVA helped to create a continuous three-dimensional network that improved the absorption capacity.^31^ The incorporation of chitosan and PVA significantly increases the number of hydrophilic units. By adding graphene oxide (GO), more functional groups are introduced, the surface area is therefore extended, and the adsorption capacity is increased, resulting in a more compact structure.^32^ This enhanced structure is the key innovation in this study. The nanocomposites’ performance was assessed in terms of heavy metal and fluoride ion adsorption under different operational conditions, along with adsorption isotherms and kinetics.

## Materials and Methods

### Materials

A high-molecular weight chitosan (CS) with a degree of deacetylation (DDA) of greater than 75% was supplied from Sigma-Aldrich Co. (Sigma-Aldrich-Merck KGaA, Darmstadt, Germany). Poly­(vinyl alcohol) (PVA) was obtained from Thermo Scientific (Waltham, MA), and kappa carrageenan (kCar) was purchased from Carl Roth GmbH & Co. KG (Karlsruhe, Germany). Acetic acid (≥99%) was obtained from Fisher Chemicals (Hampton, NH), and acetone (>99%) and glutaraldehyde (GLA) 50% in H_2_O were obtained from Sigma-Aldrich-Merck KGaA.

### Stock Solutions

Stock solutions of As­(III), Cr­(VI), and F^–^ were made by dissolving a specific amount of sodium metarsenite (AsNaO_2_), potassium dichromate (K_2_Cr_2_O_7_, ACS, ISO, Reag. Ph Eur), and sodium fluoride (NaF), respectively, in deionized water and stored. These reagents were all purchased from Sigma-Aldrich-Merck KGaA.

### Synthesis of Nanocomposites

#### Synthesis of Graphene Oxide (GO)

Graphene oxide (GO) was synthesized in the laboratory using a modified version of Marcano’s method.^33^ Actually, it is a modified version of the Hummers method^34^ that aims to improve the quality and purity of the resulting GO. Briefly, graphite is first oxidized in a mixture of sulfuric acid (H_2_SO_4_) and phosphoric acid (H_3_PO4) in a 9:1 ratio, using potassium permanganate (KMnO_4_) as the oxidizing agent. By excluding NaNO_3_ used in the Hummers method and increasing the amount of KMnO_4_, the resulting graphene oxide (GO), which is a carbonaceous material with oxygen-containing groups, provides a larger amount of hydrophilic oxidized graphene material.

#### Synthesis of CS/kCar

Initially, the CS/kCar composite was produced and evaluated for the removal of As­(III), Cr­(VI), and F^–^. For this purpose, 0.5 g of PVA was dissolved in 196 mL of doubly distilled water using a magnetic stirrer at 80 °C for 1 h. After complete dissolution, the solution was gradually cooled to 70 °C. Then, 2 g of kCar was added to the mixture, which was stirred vigorously for 15 min until the temperature decreased to 60 °C. Subsequently, 1 g of high-molecular weight chitosan was added to the well-mixed solution. To cross-link the chitosan, 4 mL of the resulting system was dissolved in a 2% acetic acid solution, followed by the addition of 1 mL of glutaraldehyde (GLA). The mixture was stirred at 60 °C for 4 h. Afterward, the composite was freeze-dried for 2 days and purified from unreacted glutaraldehyde using a Soxhlet apparatus, using an acetone/water solvent system in a 1:1 (v/v) ratio.

#### Synthesis of CS/kCar@GO

The CS/kCar@GO nanocomposite was synthesized by Marcano’s method.^33^ However, in the present study, 10 mg of GO (dissolved in 20 mL of doubly distilled water using a sonicator for 1 h) was added to the mixture before introducing the chitosan, and the solution was stirred for 10 min.

### Characterization Techniques

The structural characterization and physicochemical characterization of CS/kCar and CS/kCar@GO were conducted using advanced analytical instrumentation. Fourier transform infrared (FTIR) spectroscopy was performed with an ATR-FTIR, ZnSe spectrometer (PerkinElmer, New York, NY) to identify functional groups and chemical bonds. Crystallographic analysis was carried out using X-ray diffraction (XRD) with a Bruker (Karlsruhe, Germany) D8 FOCUS diffractometer. The surface morphology was examined via a JEOL (Tokyo, Japan) JSM6390LV scanning electron microscopy (SEM).

### Adsorption Experiments

The effect of pH was studied within the range of 3.0–9.0 over a 24 h period at a temperature of 293 K, and the determined optimal pH value remained constant for the subsequent experiments. First, 0.1 mg/L As­(III), 0.5 mg/L Cr­(VI), and 5.0 mg/L F^–^ were used as the initial concentrations, selected according to the literature and also according to the relative concentrations of each pollutant mainly found in water bodies.^35–37^ For all batch experiments, the adsorbent mass was 0.5 g/L, which is the appropriate adsorbent dosage to compare the removal efficiency, as indicated by the effect of the initial concentration (0.2–1.0 mg/L). To determine the time at which equilibrium was reached, the pollutant concentration was measured at various periods, ranging between 5 and 120 min at 293 K. The intermolecular interactions during adsorption were assessed by determining which kinetic model provided a better fit. During the isotherm experiments, various initial concentrations were tested for As­(III) (0.03–2.00 mg/L), Cr­(VI) (0.1–2.0 mg/L), and F^–^ (2.0–100.0 mg/L).

#### Adsorption Evaluation

To assess the effectiveness of the adsorbents, both removal efficiency and adsorption capacity were evaluated under various experimental conditions. The removal efficiency (*R* (percent)) of heavy metals and fluoride ions was calculated as shown in eq 1:

1 $$R\;(\%)=\frac{C_{0}-C_{e}}{C_{0}}\times 100\%$$

where *R* is the removal efficiency and *C*_0_ and *C*_e_ are the initial and equilibrium concentrations of pollutants, respectively (milligrams per liter).

The adsorption capacity was calculated as presented in eq 2:

2 $$Q_{t}=\frac{\left(C_{0}-C_{e}\right)V}{m}$$

where *Q*_t_ is the adsorption capacity (milligrams per gram), *C*_0_ and *C*_e_ are the initial and equilibrium concentrations, respectively (milligrams per liter), *m* (grams) is the weight of either CS/kCar or CS/kCar@GO, and *V* (liters) is the volume of the experiment.

#### Isothermal Studies

Numerous equilibrium models have been developed to describe the relationship between isotherms under equilibrium conditions. Among these, Langmuir^38^ and Freundlich^39^ represent the two prevailing options. The Langmuir (L) isotherm assumes monolayer adsorption,^38^ representing a homogeneous adsorption process. In contrast, the Freundlich (F) isotherm describes heterogeneous surface adsorption involving multiple layers.^39^ These models can be expressed in nonlinear form as presented in eqs 3 and 4, respectively.^38,39^ The capacities and parameters of the Langmuir and Freundlich models are crucial for understanding the adsorption behavior of emerging pollutants in various solutions.

3 $$Q_{e}=\frac{Q_{m}K_{L}C_{e}}{1+K_{L}C_{e}}$$

where *C*_e_ is the equilibrium concentration of pollutants (milligrams per liter), *Q*_m_ is the maximum sorption capacity (milligrams per gram), and *K*_L_ is the Langmuir constant (liters per milligrams).

4 $$Q_{e}=K_{f}{C_{e}}^{1/n}$$

where *n* is the heterogeneity factor and *K*_F_ is the Freundlich constant related to the adsorption capacity (units of (mg/g)·(L/mg)^1/*n*^).

#### Kinetic Study

To analyze the kinetic behavior of heavy metals and fluoride ion adsorption, the pseudo-first- and pseudo-second-order models were employed to assess the process and determine the rate-limiting step,^40^ as shown in eqs 5 and 6.

5 $$Q=Q_{e}\left(1-e^{-K_{1}t}\right)$$

6 $$Q=\frac{{Q_{e}}^{2}K_{2}t}{\left(1+Q_{e}K_{2}t\right)}$$

where *Q*_e_ and *Q* (milligrams per gram) are the adsorption capacities at equilibrium and time *t*, respectively, and *K*_1_ (inverse minutes) and *K*_2_ (grams per milligram per minute) are the first- and second-order rate constants, respectively. The adsorption process is best explained by the model with the highest correlation coefficient (*R* ^2^).

#### Adsorption Thermodynamics

Thermodynamics were studied to additionally estimate the adsorption mechanism. Thermodynamic considerations require understanding the nature of a process (i.e., spontaneous or not). The Gibbs free energy change (*ΔG*°) can be described by eqs 7 and 8.^41^

7 $$\Delta G^{{}^{\circ}}=-RT\;\ln\left(K_{c}\right)$$

8 $$\Delta G^{{}^{\circ}}=\Delta H^{{}^{\circ}}-T\Delta S^{{}^{\circ}}$$

where *R* is the gas constant (8.314 J mol^–1^ K^–1^), *T* represents the temperature (kelvin), and *K*_c_ is the thermodynamic constant.

The experimental data were obtained at 303, 313, and 323 K, using initial concentrations of 0.1 mg/L As­(III), 0.5 mg/L Cr­(VI), and 5 mg/L F^–^. The thermodynamic parameters related to adsorption, including the changes in enthalpy (Δ*H*°) and entropy (*ΔS*°), are described by eqs 9 and 10.^42^

9 $${Κ}_{C}=\frac{C_{s}}{C_{e}}$$

10 $$\ln\left({Κ}_{c}\right)=\left(-\frac{\Delta H^{{}^{\circ}}}{R}\right)+\frac{\Delta S^{{}^{\circ}}}{R}$$

#### Reuse Study

The reusability of the CS/kCar@GO composite was evaluated in batch mode using a desorbing agent. For desorption, specific quantities of CS/kCar@GO were treated with a 0.01 M NaOH solution (pH 12.0), followed by washing until reaching a neutral pH. In this case, the selected conditions allowed the pollutant to be released from the adsorbent matrix. Therefore, the NaOH solution was used for desorption. These conditions were found in the literature to be appropriate for adsorption and desorption processes of toxic heavy metals^43^ and fluoride ions,^44^ conducting regeneration experiments. Subsequently, the spent materials were reloaded with As­(III), Cr­(VI), and F^–^ to assess the composite’s life span and removal efficiency.

### Analytical Determinations

For the determination of the Cr­(VI) residual concentration, the 1,5-diphenylcarbazide (DPC) photometric method,^45^ based on the reaction of an acidified Cr­(VI) solution with 0.5% (w/v) DPC, was used. After 15 min, chromium absorption was measured at 540 nm (UV/VIS uniSPEC 4, LLG-Labware, Meckenheim, Germany) by fitting the relative absorbance to the standard curve of Cr­(VI) ions. Moreover, atomic absorption spectroscopy coupled with a graphite furnace (Varian Zeeman AA240Z with GTA 120, Palo Alto, CA) was used for the determination of the As­(III) concentration.

The SPADNS photometric method^45^ was used for the determination of fluoride ions, while a noncolored complex (ZrF_6_^2–^) was converted into a colored solution when the fluoride and zirconium anions reacted. Thus, as the fluoride concentration increased, the color of the solution became lighter. The Zr–SPADNS complex used in the analysis was prepared by mixing equal volumes of a SPADNS solution (Sigma-Aldrich-Merck KGaA) (958 mg added to 0.5 L of distilled water) and 99.99% trace element zirconyl chloride (Sigma-Aldrich-Merck KGaA) (31 mg added to 25 mL of deionized water, followed by the addition of 350 mL of HCl to a final volume of the diluted solution of 0.5 L). According to the method, 1 mL of the Zr–SPADNS reagent mixture (stable for 2 years when stored in the dark) was added to 5 mL of the sample, and the sample was mixed for 2 min. The relative absorbance at 570 nm was measured by using a UV–vis spectrophotometer (UV/vis uniSPEC 4, LLG-Labware), matching the absorbances to a standard fluoride ion curve.

### Swelling Experiments

The changes in pH influence the intermolecular interactions of adsorbent materials and determine the quantity of water that adsorbed inside the structure.^46^ The swelling ratio was measured using distilled water at pH 3.0, 5.0, and 7.0. Therefore, the composite (CS/kCar@GO) was carefully weighed (*W*_1_) (grams) and placed in water. Then, 0.05 g of the adsorbent material was added to 50 mL of each solution. Measurements were taken at specific time intervals (5, 10, 15, 30, 45, 60, 90, and 120 min) until a constant weight was achieved.

Using filter paper, the materials were blotted to remove excess surface water and then weighed (*W*_2_) (grams) until a constant weight was achieved. The swelling ratio is calculated at equilibrium using eq 11.^47^

11 $$\mathrm{swelling ratio}\;(\%)=100\%\times \frac{W_{2}-W_{1}}{W_{1}}$$

### Stability Tests

The stability of adsorbent materials was studied in distilled water at different pH values (3.0, 5.0, 7.0, 9.0, and 11.0), adjusted using HCl and NaOH solutions. A 0.05 g portion (*W*_1_) from the composite was introduced into 50 mL of the solution. Following a 24 h period, CS/kCar@GO was removed from the solution, and the samples were placed in an oven at 60 °C until they completely dried (*W*_2_) (grams). Thereafter, the adsorbent materials were weighed, and the mass loss was calculated using eq 12.^48^

12 $$\mathrm{mass loss}\;(\%)=\frac{W_{2}}{W_{1}}\times 100\%$$

## Results and Discussion

### Characterizations

#### Fourier Transform Infrared Spectroscopy (FTIR)

The chitosan spectrum features several notable bands, including a broad band between 3200 and 3500 cm^–1^ for O–H and N–H vibrations, a band at approximately 2930 cm^–1^ for aliphatic C–H stretching, and a band at 1022 cm^–1^ for C–O–C stretching, as well as another at 1651 cm^–1^ for NH_2_, which described the N–H bending vibration.^49^ The presence of sulfate groups in kappa carrageenan is indicated by absorption peaks near 923 and 840 cm^–1^.^50^ The kCar spectrum also shows a peak at 1229 cm^–1^ for S=O stretching, with additional signals at 2924 and 1046 cm^–1^ linked to C–O–C and C–H bending vibrations, respectively.^51^ The FTIR spectra of the pure materials are provided in Figure S1.

The resulting FTIR spectra of the composites are presented in Figure 1. A broad absorption band in the range of 3100–3500 cm^–1^ is observed in both spectra, corresponding to the O–H and N–H stretching vibrations of CS and kCar. The GO-containing composite (CS/kCar@GO) exhibits a broader and slightly more intense absorption in this region, indicating additional hydrogen bonding interactions between GO’s -OH, -COOH, and epoxide groups and the CS/kCar matrix.^52^

**Figure 1:**
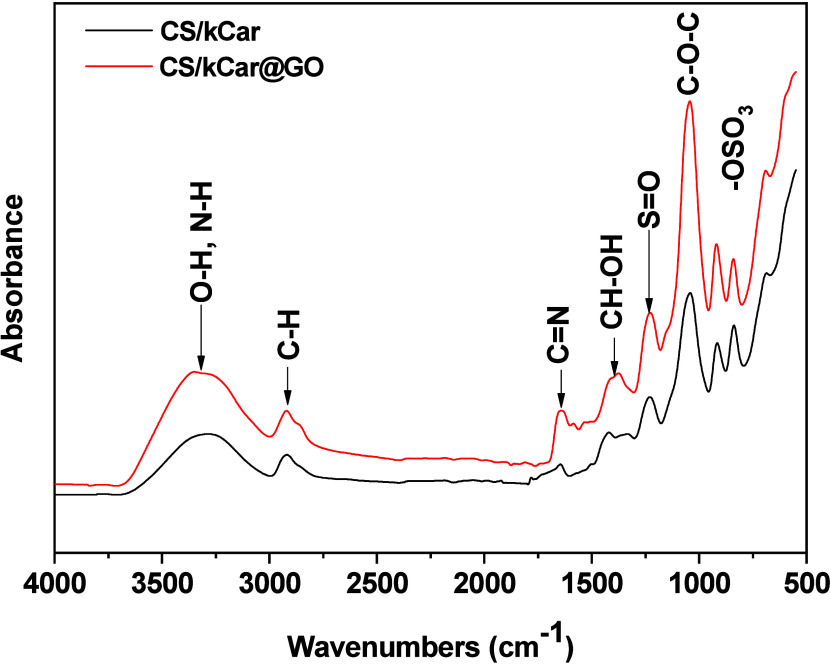
FTIR spectra of the CS/kCar and CS/kCar@GO composites.

The peaks at 1632 and 1536 cm^–1^ are assigned to the C=O stretching (amide I) and N–H bending (amide II), respectively, of chitosan that exhibits a shift in the CS/kCar@GO spectrum, suggesting enhanced interactions due to GO incorporation.^53^ Furthermore, the characteristic peak around 1640–1680 cm^–1^, attributed to C=N stretching (Schiff base formation due to GLA cross-linking), is present in both composites. The more intense and slightly shifted to a lower wavenumber peak in the GO-containing composite suggests that GO influences the cross-linking density and polymer interactions.^54^ C–O–C stretching vibrations at ∼1040 cm^–1^ are assigned to the glycosidic bonds in kCar and CS, with the intensity increased in the CS/kCar@GO composite.^55^ This observation indicates stronger intermolecular interactions and possible cross-linking reinforcement due to GO incorporation. Additionally, the peak at 1405 cm^–1^, corresponding to CH–OH vibrations, is slightly shifted, further supporting the interaction of GO with the polymeric matrix. Lastly, in the fingerprint region (500–1000 cm^–1^), the CS/kCar@GO composite exhibits more pronounced peaks and an increase in intensity, particularly in regions associated with C–O and sulfate (-OSO_3_^–^) vibrations from kCar.

After adsorption, the FTIR spectra revealed notable changes, as illustrated in Figure 2. The spectral changes indicate the involvement of key functional groups, including hydroxy (-OH), amine (-NH_2_), amide (C=O), sulfate (-OSO_3_^–^), and carboxyl (-COOH), in the adsorption process. As suggested by the results, the pollutants do not interact directly with the O–H groups in any of the cases, leading to the decrease in the peak’s intensity. On the contrary, Cr­(VI) and As­(III) can form outer-sphere complexes due to electrostatic interactions with O–H groups without breaking them.^56^ Thus, instead of reducing the number of O–H signals, these interactions increase the level of hydrogen bonding between hydroxy groups and adsorbed ions, making the O–H band appear more pronounced. This effect is especially strong in GO-containing composites, where hydroxy and carboxyl groups form extensive hydrogen bond networks.^57^ This observation is more profound for Cr­(VI) adsorption (Figure 2b,e), where a shift in the O–H and N–H stretching region (∼3400 cm^–1^) occurs, accompanied by changes in the amide I (∼1632 cm^–1^) and amide II (∼1536 cm^–1^) peaks. Except for the broadening of the existing O–H band, an extremely weak and irregular absorption feature is observed after adsorption, possibly being an overtone band of As adsorption effects (Figure 2a,d). Additionally, a reduction in the intensity of the sulfate (-OSO_3_^–^) peak (∼1220–1000 cm^–1^) supports this finding. The successful adsorption of Cr­(VI) and As­(III) is indicated by the sharpening of peaks in the fingerprint region and even the formation of new ones (750–800 cm^–1^), which was observed in the case of As (for both composites).^58^

**Figure 2:**
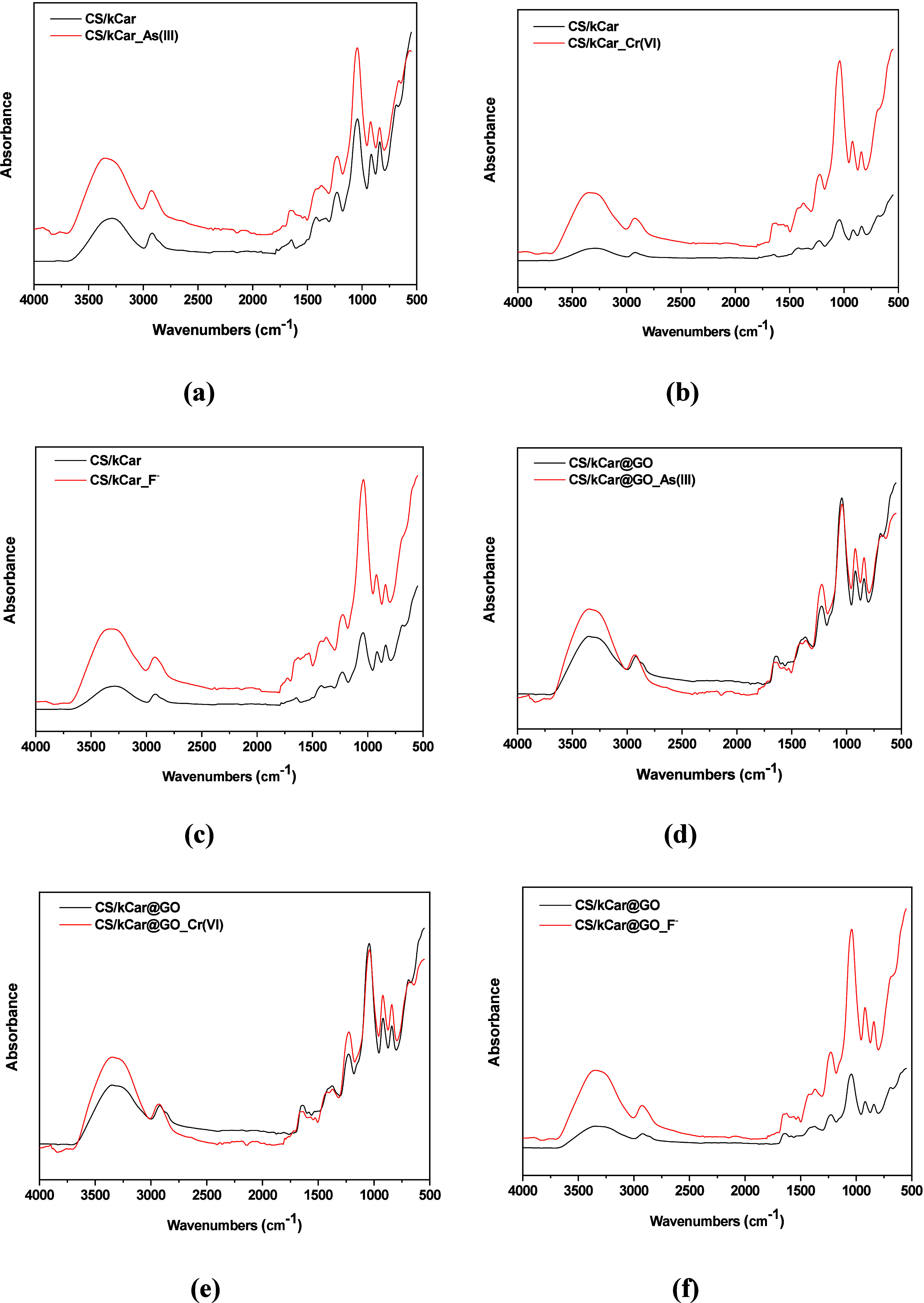
FTIR spectra after adsorption onto CS/kCar for (a) As­(III), (b) Cr­(VI), and (c) F^–^ and onto CS/kCar@GO for (d) As­(III), (e) Cr­(VI), and (f) F^–^.

Additionally, the data from the FTIR spectrum indicated variations in the absorbance peaks for F^–^ (Figure 2c,f), with peaks occurring between 1635 and 1655 cm^–1^. This observation strongly supports the functional interaction between the composites and pollutants, likely through ion–dipole interactions.^59^ The broadened peaks are linked to characteristic amino groups, suggesting that nitrogen atoms with nonbonding electron pairs are engaging with the pollutant anions.^60^

#### X-ray Diffraction (XRD)

The structure, composition, and purity of the prepared materials were analyzed using an XRD characterization technique. Figure 3 illustrates the patterns of pure CS, pure kCar, and the CS/kCar and CS/kCar@GO composites.

**Figure 3:**
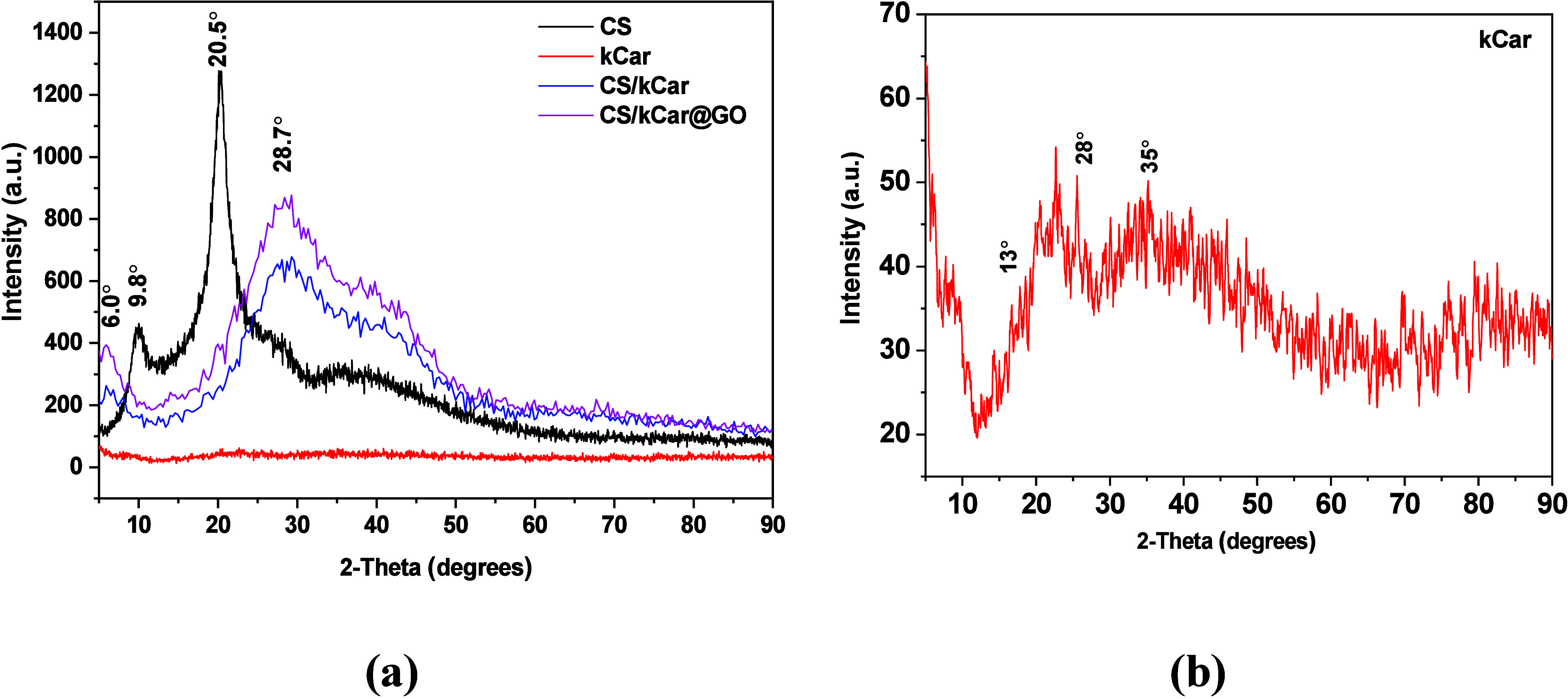
(a) XRD of CS, pure kCar, CS/kCar, and CS/kCar@GO composites. (b) Close-up of kCar XRD.

The X-ray diffraction patterns presented in Figure 3 provide insight into the interactions of the components occurring in the composite. First, CS in its pure form presents two characteristic peaks at 9.8° and 20.5° corresponding to its semicrystalline nature.^61^ The neat kCar (better view in Figure 3b) shows broad and low-intensity peaks from ∼13° to ∼28° and a second one with its maximum at 35° indicating its amorphous and possibly hydrated nature.^62^ The composite now of those two components gives rise to a small peak at 6°. This peak is explained by the contribution of the cross-linker used (GLA) and its helix formation due to polymer–polymer interactions.^63^ Another possible explanation is the expansion of the polymer chain of CS due to interactions with kCar and GLA that shift its 9.8° peak to a smaller 2θ (6°) ascribed to a larger *d* spacing.^64^ A broader peak at 28.5° (increasing from 14° to 55°) of the composites is ascribed to the formation of a polyelectrolyte complex by the positively and negatively charged CS and kCar, respectively.^65^ It seems that GO was embedded within the composite’s matrix; thus, no characteristic peak (∼10°) of GO is observed for CS/kCar@GO, but the slightly higher intensity of the latter indicates that the presence of GO in the composite matrix enhances the crystallinity.^66^

#### SEM Analysis

The microstructure of CS/kCar and CS/kCar@GO aerogels was examined by using SEM. Figure 4a is the micrograph of CS/kCar where the layers of the aerogel showcase a wrinkled surface that is expected when CS interacts electrostatically or forms hydrogen bonds with other polymers.^67^ Furthermore, alterations in the hydrogel network during dehydration process may lead to the development of wrinkles, too.^68^ On the other hand, CS/kCar@GO presents a smoother surface indicative of a good distribution of GO within the matrix.^69^

**Figure 4:**
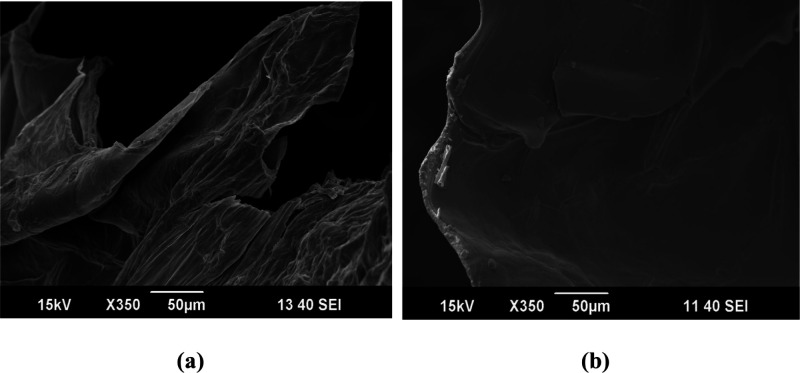
SEM images of (a) CS/kCar and (b) CS/kCar@GO.

### Adsorption Evaluation

#### pH Effect

The pH of a solution is a crucial factor that defines all other aspects of adsorption.^70^ The relative impact on the removal efficiency (percent) of As­(III), Cr­(VI), and F^–^ is illustrated in Figure 5. As one can see, the CS/kCar@GO composite achieved the highest removal efficiency for all pollutants, while the neat kCar exhibited the lowest removal efficiency. The results revealed that the maximum removal for As­(III) and fluoride ions was achieved at pH 5.0 and 3.0, respectively. In particular, 79.3% of As­(III) at pH 5.0 and 35.8% of F^–^ at pH 3.0 were achieved by applying 0.5 g/L CS/kCar@GO, while the other two materials, kCar and CS/kCar, presented even less than 20% removal efficiency. Regarding Cr­(VI), it is observed that its removal, mainly by CS/kCar@GO, is less dependent on pH, exhibiting more than 98% Cr­(VI) removal over the whole pH range. Therefore, for the next experiments, pH 7.0 is selected, as it is closer to water’s pH in practice. This is very important as at the pH closest to the value of surface and groundwater, it is also effective and does not require any pH adjustment.

**Figure 5:**
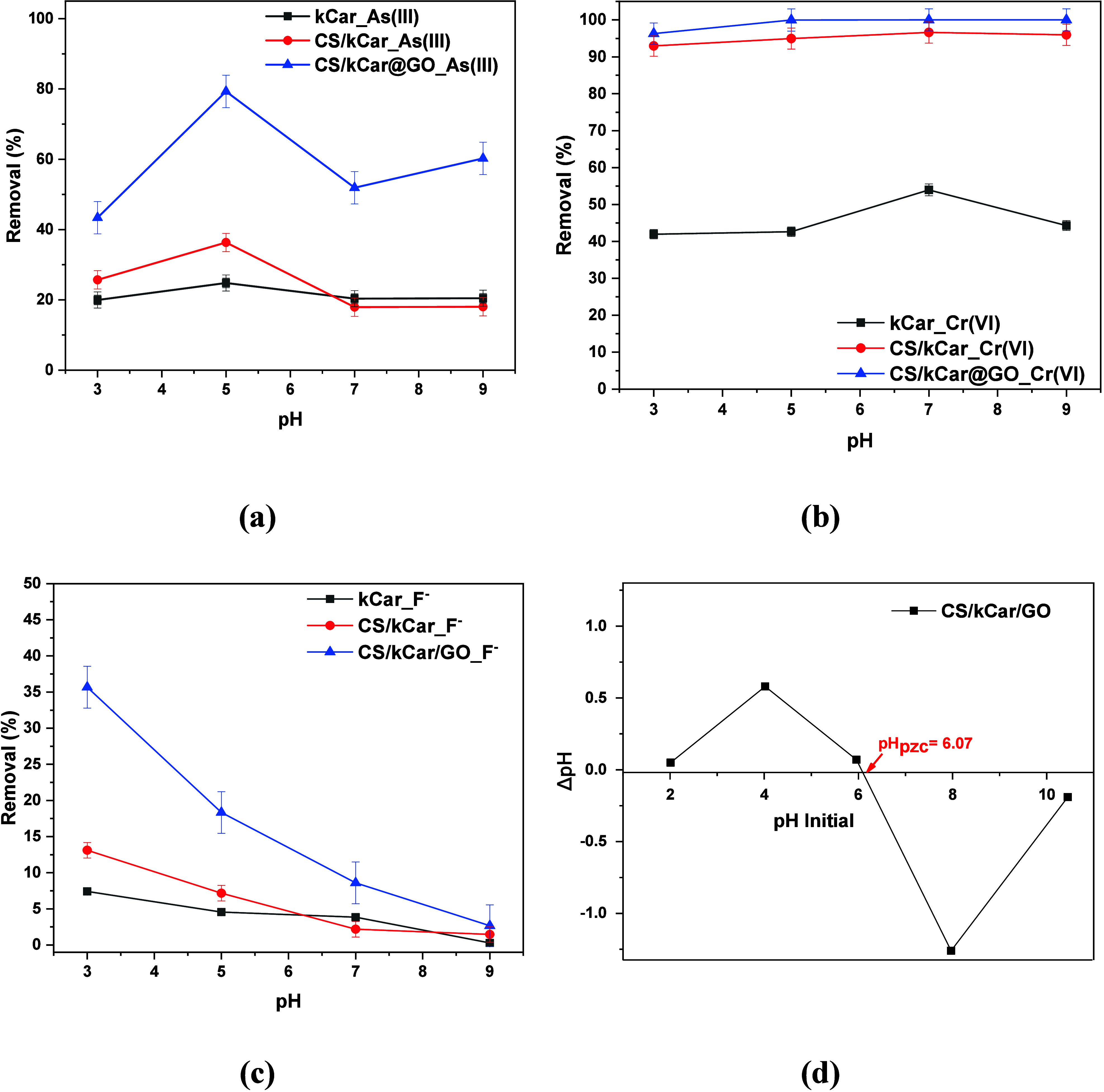
Effect of pH on the adsorption of (a) As­(III), (b) Cr­(VI), and (c) F^–^. The experiments were performed with initial contaminant concentrations of 0.1 mg/L As­(III), 0.5 mg/L Cr­(VI), and 5 mg/L F^–^. The adsorbent dose was 0.5 g/L, with pH values of 3.0, 5.0, 7.0, 9.0, and 11.0 and a contact time of 24 h, and the temperature was maintained at 293 K. (d) pH_pzc_ of the CS/kCar@GO adsorbent.

Moreover, Figure 5d shows that the point of zero charge (pH_pzc_) of the optimum CS/kCar@GO material was measured by applying the pH drift method^71^ and found to be 6.07. Therefore, when pH > pH_pzc_ the surface is positively charged and when pH < pH_pzc_ the surface becomes negatively charged. Thus, at optimum pH values of 5 for As­(III) and 3 for F^–^ (<pH_pzc_), the material has the ability to attract arsenic^72^ and fluoride^73^ ions via electrostatic interaction. In the case of Cr­(VI), negative anions present under acidic conditions, i.e., HCrO_4_^–^ and Cr_2_O_7_^2–^,^74^ may latch onto the surface of the adsorbent via electrostatic interactions. Above pH 7, exceeding the pH_pzc_, there is a repulsion leading to a competition between Cr_2_O_4_^2–^ and OH^–^.

Based on the above, a possible interaction between the CS/kCar@GO adsorbent and As­(III), Cr­(VI), and F^–^, that might take place, depending on each individual pollution, is illustrated in Figure 6. Specifically, the adsorption of As­(III), Cr­(VI), and fluoride ions in CS/kCar@GO occurs through different mechanisms, which depend on the interactions between the pollutants and the functional groups of the materials. First, As­(III) is mainly adsorbed via electrostatic interactions, under optimum pH conditions because As­(III) interacts with the hydroxy and carboxyl group of CS/kCar@GO. Additionally, chitosan may interact with As­(III) through electrostatic and hydrogen bonding interactions, particularly with the positively charged protonated amino groups on the chitosan polymer.^75^ Moreover, the adsorption of Cr­(VI) takes place via hydrogen bonds, where the functional group of CS/kCar@GO (carboxyl and amino groups) interacts with Cr­(VI), forming intermolecular interactions. These interactions play a role in the removal and detoxification of Cr­(VI) from aqueous solutions. In addition, the amine and hydroxy groups on the chitosan molecule act as binding sites for Cr­(VI) ions, particularly at low pH, where the amine groups become protonated and positively charged, enhancing electrostatic interactions. Some studies also suggest a reduction mechanism, where chitosan, or modified chitosan-based materials, can facilitate the reduction of Cr­(VI) to a less toxic form.^76^

**Figure 6:**
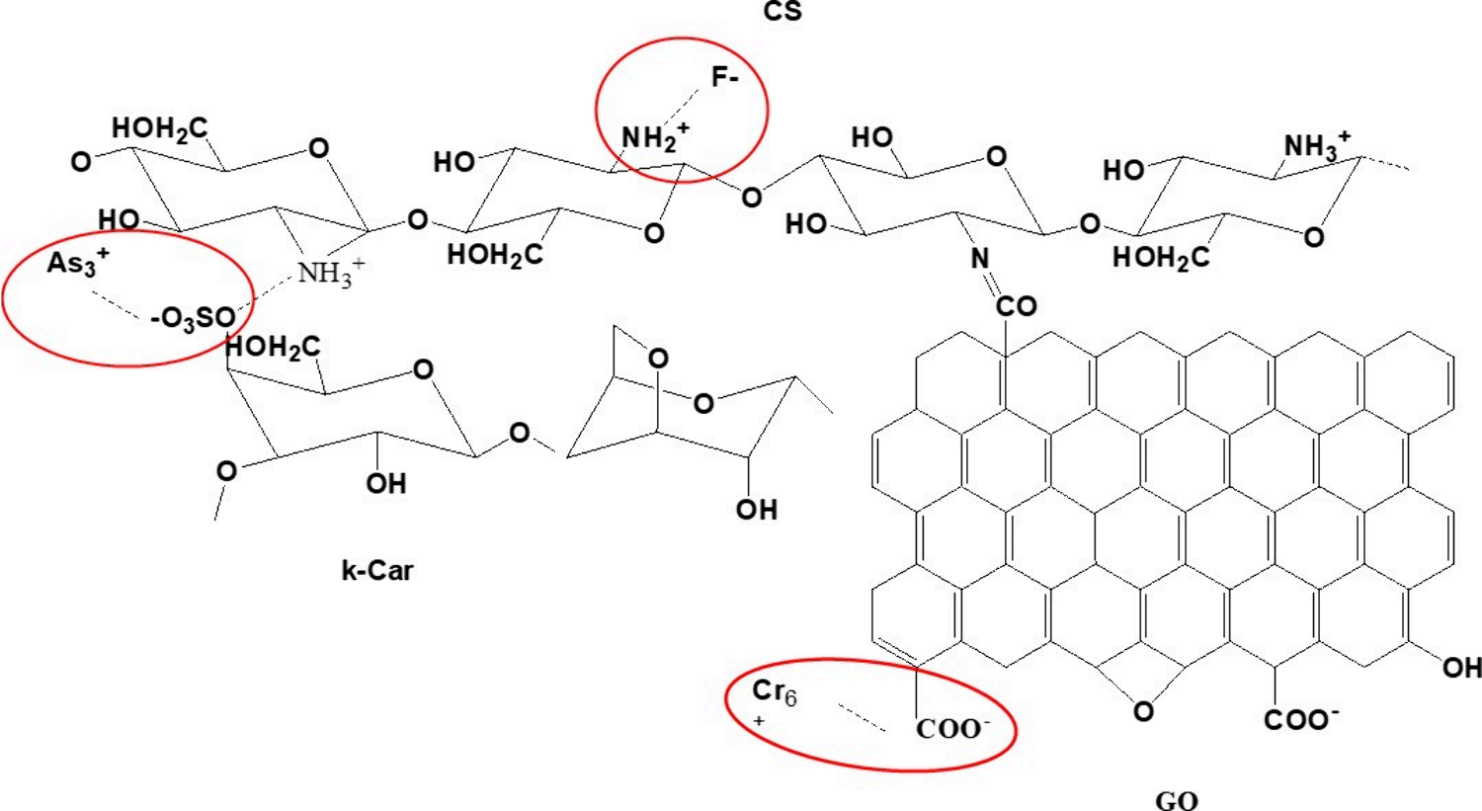
Possible interactions between the CS/kCar@GO adsorbent and As­(III), Cr­(VI), and F^–^.

The adsorption of fluoride ions is mainly based on ion–dipole interactions, where the negatively charged F^–^ interacts with the polarized functional group on the surface area of CS/kCar@GO, such as an amino group. This interaction is driven by electrostatic attraction and ion exchange between the positively charged amino groups of chitosan and the negatively charged fluoride ions.^77^ However, the FTIR analysis (see X-ray Diffraction (XRD)) confirmed the existence of these interactions, as a change in the characteristic vibrations of the functional groups was observed after adsorption indicating a chemical interaction between the pollutants and adsorbent material.

These findings underscore the critical role of pH in optimizing the adsorption process and highlight the enhanced performance of the CS/kCar@GO composite compared to other materials. The highest removal efficiency for diverse pollutants makes CS/kCar@GO a promising candidate for advanced water treatment applications.

#### Swelling Study

The degree of swelling depends on the pH values.^78^ An increase in the number of hydrophilic groups enhances the formation of the hydrogen bond.^79^ Chitosan contains functional groups such as -OH and -NH_2_, which are protonated under acidic conditions. Moreover, kCar is degraded at low pH,^80^ while graphene oxide contains various functional groups, such as -OH and -COOH, which are protonated at low pH. Therefore, at pH 3.0 and 5.0, the CS/kCar@GO adsorbent acquires a positive charge, thereby promoting the formation of hydrogen bonds with water molecules. Figure 7a shows the swelling percentage of the best nanocomposite, that of highest adsorption capacity (CS/kCar@GO) at pH 3.0 (optimum found for fluoride ion removal), pH 5.0 (optimum found for As­(III) removal), and pH 7.0 (optimum found for Cr­(VI) removal).

**Figure 7:**
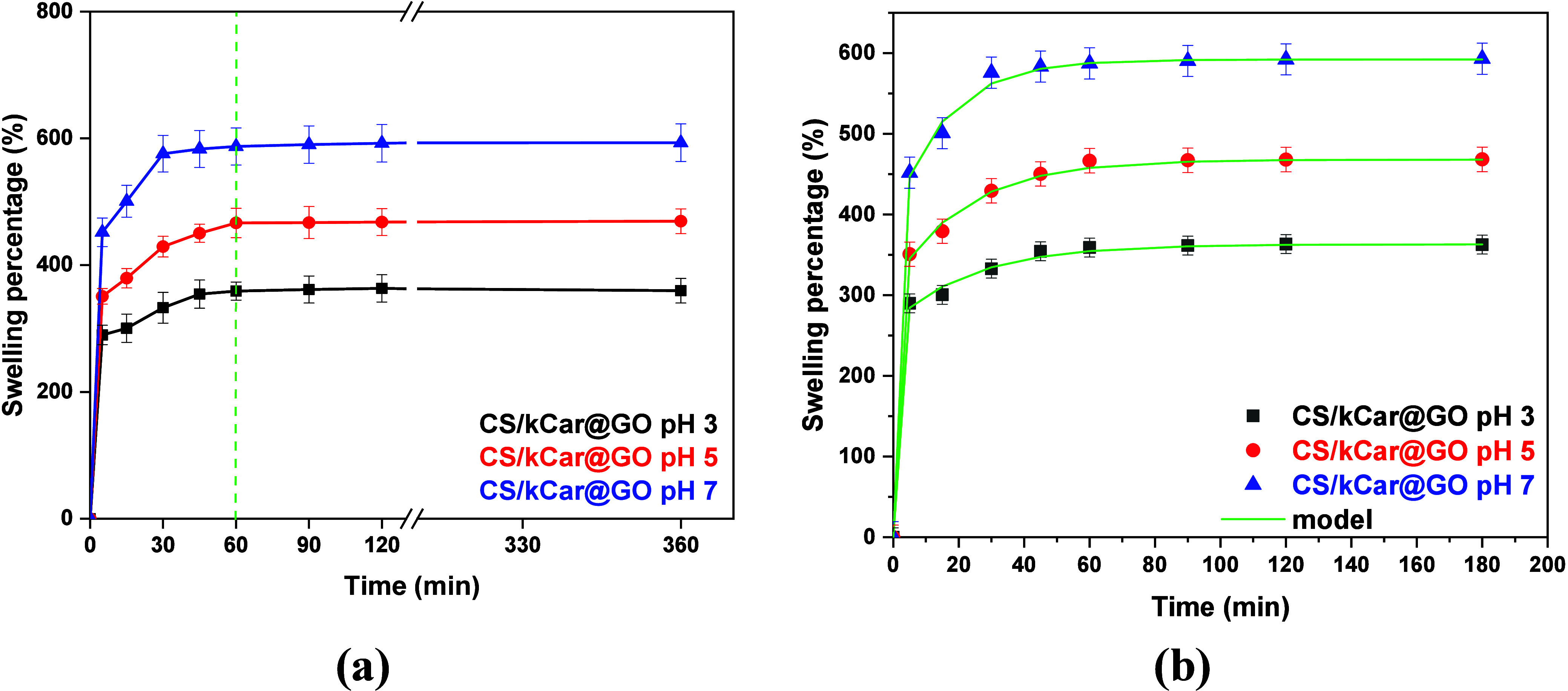
(a) Experimental swelling percentage of CS/kCar@GO at pH 3.0, 5.0, and 7.0. (b) Experimental and fitted evolution of swelling for CS/kCar@GO and three pH values.

As Figure 7a shows, the swelling percentage increases with pH. This is attributed to the interactions of the functional groups with water molecules taking into consideration the p*K*_a_ (under neutral conditions the water molecules can more easily enter the composite’s network).^78^ The neutral pH values favor the entrance of water molecules on the composite’s network due to the absence of repulsive forces between the water molecules and the functional groups of the material. Also, as swelling continues, the water molecules continue to penetrate the composite’s network, but after a crucial time (60 min for all pH values), the swelling reaches a plateau. It appears that swelling is a two-stage process. A first fast step is followed by a second, much slower step. Let *S* denotes the swelling percentage. The appropriate functional form for the two-stage process is shown in eq 13:

13 $$S=S_{o}\left[1-\varphi \;\exp\left(-s_{1}t\right)-\left(1-\varphi \right)\;\exp\left(-s_{2}t\right)\right]$$

where *S*_o_ is the asymptotic percentage swelling, φ is the fraction of water entering the matrix with kinetic constant *s*_1_, and 1 – φ is the fraction of water entering the matrix with kinetic constant *s*_2_. A least-squares fitting process leads to estimation of the above parameters. The comparison between experimental data and fitting curves is shown in Figure 7b. The calculated values of the parameters are listed in Table 1.

**Table 1:** Estimated Parameters of the Swelling Model for CS/kCar@GO and Three pH Values

	*S*_o_ (%)	φ	*s*_1_ (min^–1^)	*s*_2_ (min^–1^)
pH 3.0	363	0.27	0.04	3.13
pH 5.0	468	0.33	0.045	3.34
pH 7.0	592	0.33	0.063	3.34

It appears there is a huge difference between the two kinetic constants (about 2 orders of magnitude) and the larger fraction of water enters the matrix with the higher kinetics. A close inspection to the fitting results reveals that only the asymptotic swelling is a strong function of pH. The other constants vary slightly with pH, and the assumption of the independence of pH values would marginally decrease the quality of the fitting.

#### Contact Time Effect

The contact time is a key parameter for assessing the kinetics of the adsorption process.^81^ To examine this effect for As­(III), Cr­(VI), and F^–^, various contact times were examined, i.e., 10, 30, 60, 90, and 120 min. Figure 8 illustrates the relationship between the adsorption and contact time. Initially, as one can see, all of the adsorption sites on the adsorbent material are noncovered. As the contact time increases, interactions lead to an increase in the uptake of As­(III), Cr­(VI), and F^–^ until equilibrium is reached after 30 min for As­(III) and Cr­(VI) removal and 100 min for F^–^. After this time, there is no further increase, as the adsorbent held the maximum amount of pollutants.^82^

**Figure 8:**
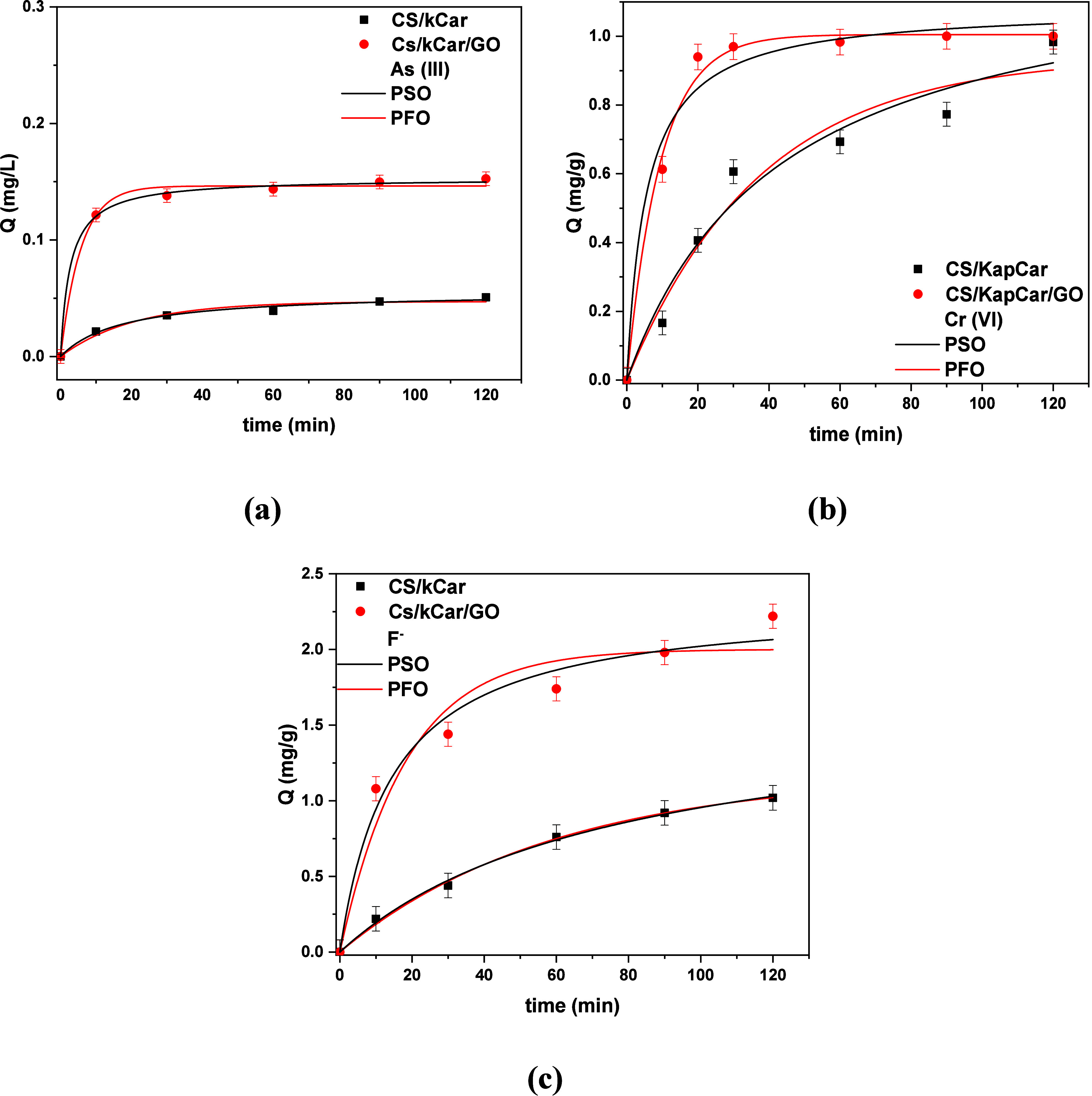
Effect of contact time on the adsorption of (a) As­(III), (b) Cr­(VI), and (c) F^–^. The experiments were performed with initial contaminant concentrations of 0.1 mg/L As­(III) at pH 5.0, 0.5 mg/L Cr­(VI) at pH 7.0, and 5 mg/L F^–^ at pH 3.0. The adsorbent dose was 0.5 g/L, with contact times of 10, 30, 60, 90, and 120 min. The temperature was maintained at 293 K.

A critical factor that influences the adsorption process, as the rate and mechanism of adsorption can be inferred from it, is the adsorpti on kinetics. The results aligned with the PSO model, indicating a tendency toward chemosorption for the removal of all examined materials (higher correlation coefficient (*R* ^2^)), and the relative determined parameters are listed in Table 2.

**Table 2:** PFO and PSO Kinetic Model Parameters for As­(III), Cr­(VI), and F^–^

			PFO			PSO		
pollutant	adsorbent	*Q*_e,exp_ (mg/g)	*K*_1_ (min^–1^)	*Q*_e,cal_ (mg/g)	*R* ^2^	*K*_2_ (L mg^–1^ min^–1^)	*Q*_e,cal_ (mg/g)	*R* ^2^
As(III)	CS/kCar	0.73	0.005	0.047	0.968	1.017	0.056	0.987
	CS/kCar@GO	0.16	0.174	0.14	0.993	2.393	0.15	0.998
Cr(VI)	CS/kCar	0.97	0.026	0.93	0.961	0.024	0.93	0.997
	CS/kCar@GO	1.00	0.105	0.10	0.993	1.254	1.00	0.996
F^–^	CS/kCar	1.32	0.016	1.17	0.975	1.084	1.68	0.993
	CS/kCar@GO	3.56	0.054	2.00	0.946	0.029	2.31	0.977

On the other hand, the empirical adsorption kinetic models, such as the pseudo-first- and pseudo-second-order models, have been extensively used for the analysis of experimental adsorption data. Traditionally, the pseudo-second-order model has been shown to fit the data excellently. According to Simonin^83^ and Kostoglou and Karapantsios,^84^ this success is not essential but is due to the linearization of the fitting process. This is why the nonlinear fitting process was used in the present work. However, even if it succeeds in fitting, the empirical models have other important drawbacks. The main drawbacks of these methods are that they do not take into account the solute mass balance, they cannot be used to scale up the process, and their parameters do not have a direct physical meaning. The above issues can be overcome using a phenomenological model. Even a highly approximating phenomenological model can offer more information than empirical models.

The adsorption by hydrogels and aerogels is very difficult to model in a phenomenological way. Typically, the empirical modeling is used.^85^ The main problem is swelling and its competition with adsorption. In some cases, swelling occurs much faster than adsorption/desorption and the two processes can be studied as the one isolated from the other.^86^ In the present case, adsorption occurs on the same time scale as swelling. Such a problem has been studied previously by Kyzas et al.,^87,88^ as they found that the characteristic times of two phenomena, i.e., swelling and adsorption, are comparable, so the swelling state of the adsorbent particle affects adsorption kinetics. In particular, a mixed kinetic behavior for the swelling of the hydrogel has been considered. A part of the adsorbent water is distributed uniformly in the adsorbent particle, and a part is adsorbed at the saturation fraction in the outer shell of the particle. The relative amounts of water are determined by using experimental data of adsorption. However, in order to follow this procedure, adsorption data for different degrees of swelling of the adsorbent at the beginning of adsorption are needed. This is not the case here where only data for an initially dry adsorbent exist, so an elaborated model like those in refs 87 and 88 cannot be employed.

The particles of the adsorbent in the present work are in the shape of flakes. The two sides have sizes on the order of centimeters, and the third side has a size on the order of tenths of micrometers. Therefore, the aspect ratio is very large, and the adsorption process can be considered to occur in a slab. The adsorption mechanism of heavy metals by aerogels can be due to several reasons;^89^ however, it is believed that the kinetics is determined by a diffusion step. The effect of swelling cannot be assessed in the absence of adsorption data under several degrees of swelling, so an assumption has to be made. The assumption is that the decrease in diffusion kinetics due to space expansion is counterbalanced by the increase due to the higher water content. Let us proceed with a mathematical model for diffusion in slab geometry. The local concentration of adsorbed material at distance *x* from the center of the slab is denoted as *q*(*x*). The evolution of *q*(*x*) is determined from eq 14:

14 $$\frac{\partial q}{\partial t}=D\frac{\partial^{2}q}{\partial x^{2}}$$

where *D* is the diffusivity of the solute in the adsorbent particle. The initial condition at time zero is *q* = 0. The boundary conditions are zero flux at *x* = 0 due to symmetry and *q*(*L*) = f­(*C*). The second is the equilibrium condition at the interface between the adsorbent and water phase. *L* is denoted as the half-thickness of the slab, and *f*(*C*) denotes the Langmuir adsorption isotherm. The average adsorption load is computed with eq 15:

15 $$Q=\frac{1}{L}\int_{0}^{L}q\;dx$$

Finally, the problem is solved by considering the solute mass balance *C* = *C*_o_ – *mQ*/*V*, where *C*_o_ is the initial solute concentration, *m* is the adsorbent mass, and *V* is the liquid volume. The mathematical problem contains a partial differential equation, and it must be solved numerically. In order to simplify the problem, a quadratic polynomial profile is assumed for *q*(*x*). Application of the boundary conditions to the assumed profile indicates that it must have the form of eq 16:

16 $$q=\alpha \left(x^{2}-L^{2}\right)+f\left(C\right)$$

where α is a function of time that has to determined. Taking the integral of eq 14 from *x* = 0 to *L* and dividing by *L* lead to eq 17:

17 $$\frac{\partial Q}{\partial t}=\frac{D}{L}{\left(\frac{\partial q}{\partial x}\right)}_{x=L}=2\alpha D$$

Substitution of eq 15 into eq 16 leads to eq 18:

18 $$Q=-\frac{2\alpha L^{2}}{3}+f\left(C\right)$$

Finally combining eqs 17 and 18 leads to the following ordinary differential equation (eq 19) for the evolution of *Q*, which must be solved combined with the solute mass balance:

19 $$\frac{dQ}{dt}=\frac{3D}{L^{2}}\left(f\left(C\right)-Q\right)$$

The above equation is solved numerically in combination with the mass balance, and the calculated evolution of *C* is fitted to the experimental adsorption data.

The corresponding fits to the experimental data appear in Figure 9 for As­(III), Cr­(VI), and F^–^. The values of *D*/*L* ^2^ found from the fit are listed in Table 3.

**Figure 9:**
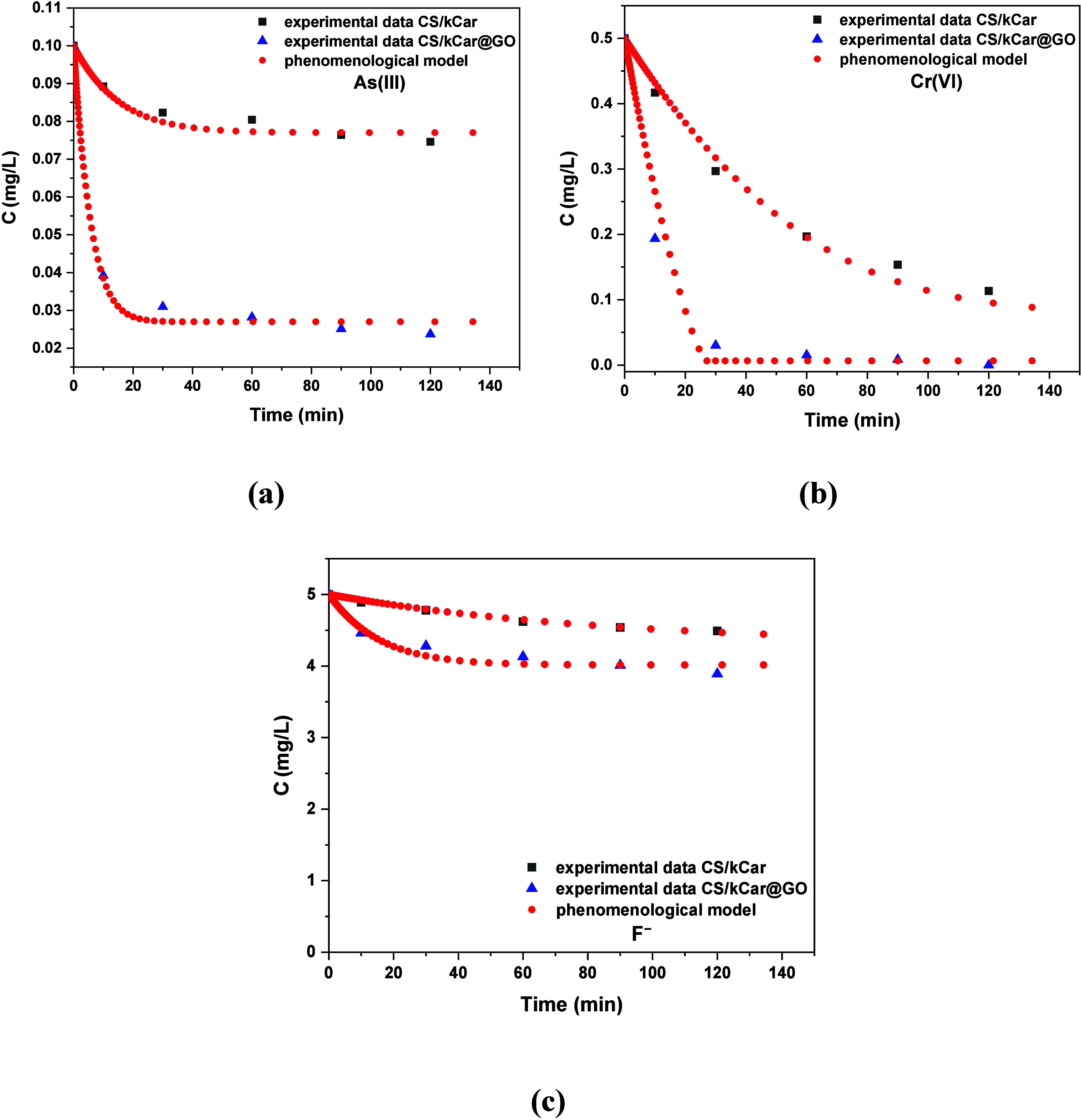
Comparison of the evolution of the bulk concentration of (a) As­(III), (b) Cr­(VI), and (c) F^–^ between the experiment and model. Results were for adsorbents CS/kCar and CS/kCar@GO.

**Table 3:** *D*/*L* ^2^ Ratios (inverse minutes) That Resulted from Fitting the Experimental Adsorption Kinetic Data

	*D*/*L*^2^ (min^–1^)		
	As(III)	Cr(VI)	F^–^
CS/kCar	0.017	0.0020	0.0033
CS/KCar@GO	0.017	0.0066	0.0170

It appears that the diffusivity for As­(III) (Figure 9a) is the same for the two adsorbents whereas for the other two ions (Figure 9b,c) it is much larger in the presence of graphene oxide. It is noted that unlike the pseudo-second-order model, the present model is capable of isolating the effect of driving force (distance from equilibrium) from purely kinetic aspects (such as the diffusion constant) on the experimental adsorption data. In order to get an idea of the meaning of the above values, a calculation for the diffusivity of hydrated but not swollen material can be found by assuming roughly *L* = 25 μm. The diffusion coefficient for As­(III) can be calculated to be 1.77 × 10^–13^ m^2^/s.

#### Adsorption Isotherms

Two isotherm models were applied to successfully describe the adsorption process. The correlation coefficient (*R* ^2^) values indicated that similarly, the Langmuir and Freundlich isotherm models both fitted the experimental data (Table 4). According to the Freundlich model,^39^ the nanocomposite exhibited saturation with As­(III), Cr­(VI), and F^–^ ions. Simultaneously, the Langmuir model suggested^90^ the formation of monolayer adsorption on the nanocomposite surface. Among the models evaluated, the Langmuir model provided the best fit for simulating the adsorption of the studied pollutants, as evidenced by its highest correlation coefficient. This model demonstrated that the ratio of the compound concentration remaining in solution to the concentration adsorbed on the solid decreased as the solute concentration increased, producing a concave curve indicative of progressive solid saturation. The adsorption of As­(III), Cr­(VI), and F^–^ onto CS/kCar@GO was identified as a homogeneous process primarily driven by chemical adsorption. Using the Langmuir isotherm equation (Figure 10), the maximum adsorption capacities (*Q*_m_) were calculated to be 2.44 mg/g for As­(III), 2.82 mg/g for Cr­(VI), and 32.63 mg/g for F^–^ with the optimum CS/kCar@GO. The superior uptake of F^–^ was attributed to its lower hydration number, which facilitates its attachment to the nanocomposite surface.^91^ Additionally, an *R*_L_ value between 0 and 1 verified the spontaneity of the adsorption process.

**Figure 10:**
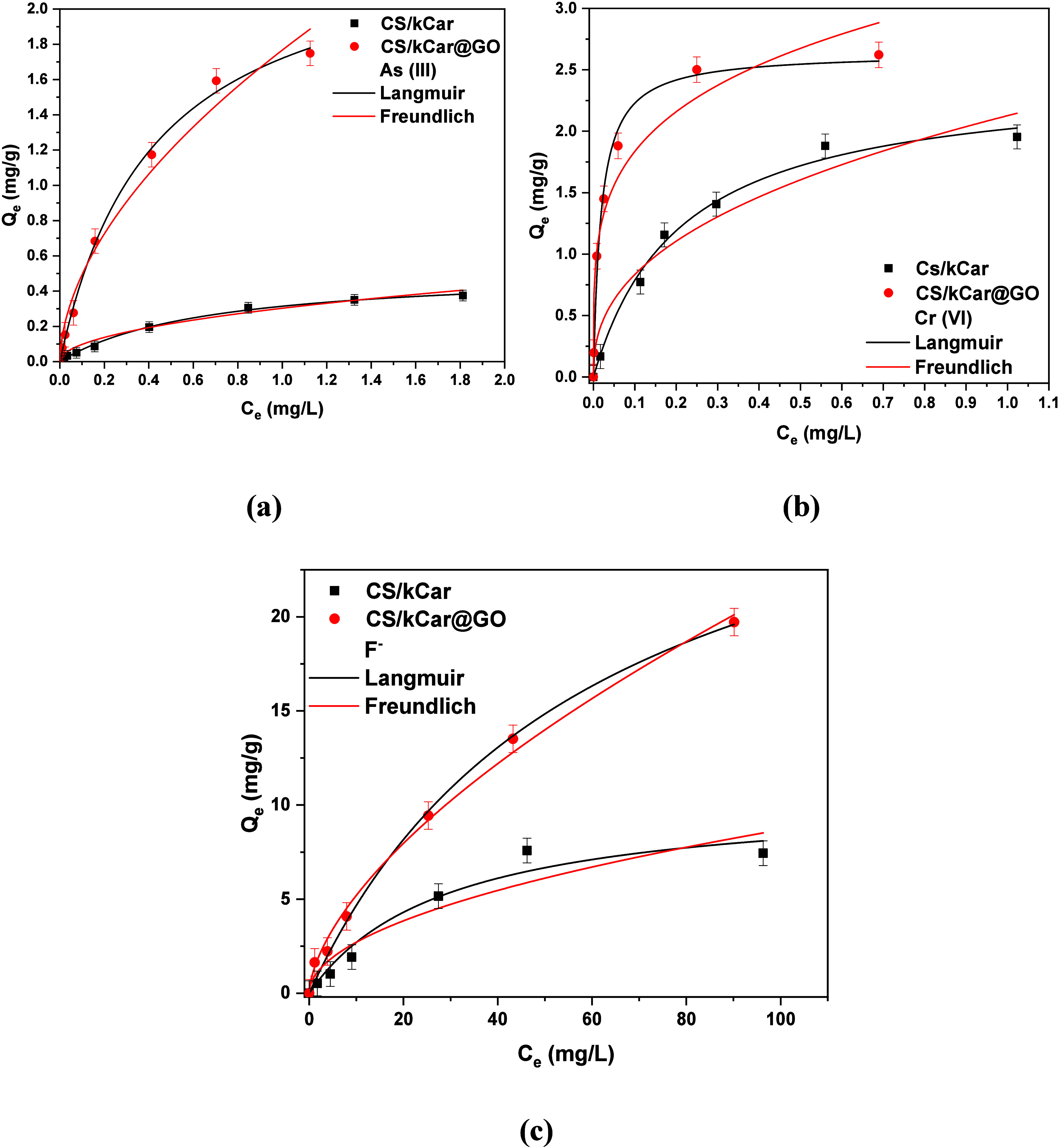
Fitting of the Langmuir and Freundlich isotherm models.

**Table 4:** Constants of Isotherm Models for As­(III), Cr­(VI), and F^–^ Adsorption Materials

		Langmuir model			Freundlich model		
pollutant	adsorbent	*K*_L_ (L/mg)	*R* ^2^	*Q*_m_ (mg/g)	*K*_F_ (mg/g)(L/mg)^1/n^	*R* ^2^	1/*n*
As(III)	CS/kCar	1.50	0.997	0.52	0.303	0.981	0.62
	CS/kCar@GO	2.38	0.998	2.44	1.767	0.979	0.55
Cr(VI)	CS/kCar	4.76	0.991	2.45	2.127	0.943	0.41
	CS/kCar@GO	54.12	0.990	2.82	3.145	0.925	0.23
F^–^	CS/kCar	0.034	0.966	10.53	0.845	0.895	0.51
	CS/kCar@GO	0.016	0.996	32.63	1.261	0.991	0.62

The Freundlich constant (1/*n*) reflected the adsorption intensity, categorized as follows: 0.1 < 1/*n* ≤ 0.5 (very easy to adsorb), 0.5 < 1/*n* ≤ 1 (easy to adsorb), and 1/*n* > 1 (difficult to adsorb).^92^ For all pollutants, the 1/*n* values did not exceed 1, indicating favorable adsorption conditions. In particular, the 1/*n* values were 0.55 for As­(III) and 0.62 for F^–^, indicating easy adsorption, while Cr­(VI) presented a 1/*n* value of 0.23, suggesting that it was very effective.

#### Adsorption Thermodynamics

Several thermodynamic parameters were investigated during the adsorption process (Table 5). Since *ΔG*° is negative for heavy metals at all tested temperatures, i.e., 303, 313, and 323 K, it can be concluded that the adsorption of As­(III), Cr­(VI), and F^–^ by the CS/kCar@GO nanocomposite was spontaneous.

**Table 5:** Parameters of Isotherms for the Adsorption of As­(III), Cr­(VI), and F^–^ onto CS/kCar@GO

	*T* (K)	Δ*G*° (kJ/mol)	Δ*H*° (kJ/mol)	Δ*S*° (kJ mol^–1^ K^–1^)	*R* ^2^
As(III)	303	–3.120	12.861	0.053	0.979
	313	–3.647			
	323	–4.175			
Cr(VI)	303	–2.700	19.111	0.072	0.932
	313	–3.420			
	323	–4.140			
F^–^	303	–0.128	19.717	0.066	0.976
	313	–0.783			
	323	–1.438			

The positive values of *ΔS*° of CS/kCar@GO are 0.053, 0.072, and 0.066 kJ mol^–1^ K^–1^ for As­(III), Cr­(VI), and F^–^, respectively, suggesting an increased level of randomness at the solid–liquid interface, potentially due to the release of water molecules during the adsorption process.^93^ Additionally, the positive Δ*H*° values of 12.81, 19.11, and 16.717 kJ/mol indicate endothermic adsorption processes.

#### Regeneration Study

The regeneration of adsorbents is essential for environmental sustainability, enabling the reuse of materials in water purification processes and thus decreasing both costs and waste production.^94^ Figure 11 shows the adsorption performance of CS/kCar@GO for As­(III), Cr­(VI), and F^–^ after 10 recycling cycles. As the number of cycles increased, the adsorption capacities for As­(III), Cr­(VI), and F^–^ gradually decreased, as expected.

**Figure 11:**
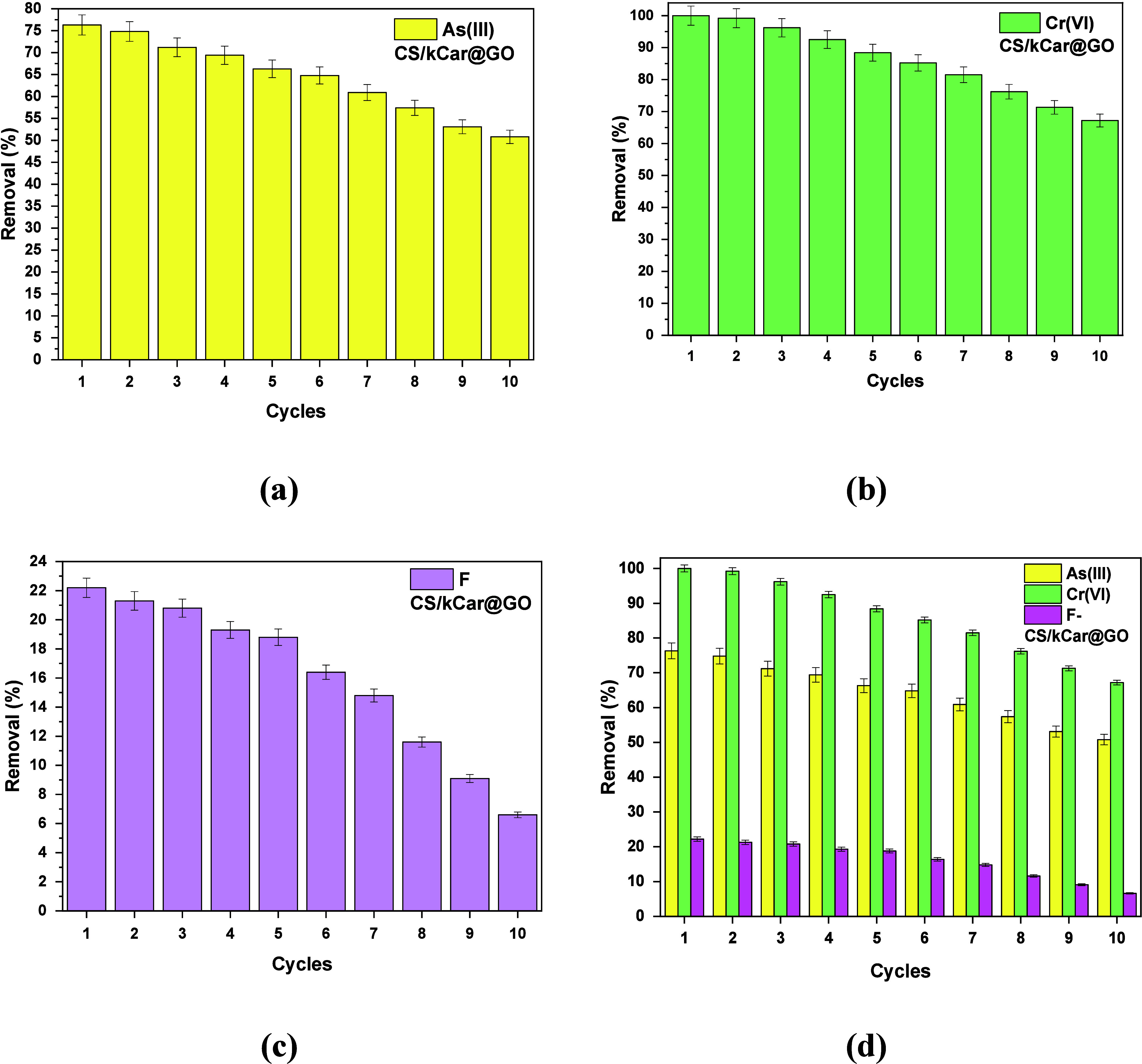
Adsorption of (a) As­(III), (b) Cr­(VI), and (c) F^–^ onto CS/kCar@GO and (d) merged chart with all pollutants for 10 cycles after regeneration at pH 10 using 0.1 M NaOH treatment: 0.1 mg/L As­(III) at pH 5.0, 0.5 mg/L Cr­(VI) at pH 7.0, and 5 mg/L F^–^ at pH 3.0 for 2 h.

The reusability of CS/kCar@GO for the removal of As (III), Cr­(VI), and F^–^ was investigated through adsorption–desorption experiments. The material could be reused effectively for up to 10 cycles, for As­(III), Cr­(VI), and F^–^ removal, after successful regeneration by 0.01 M NaOH. In the first cycle, the removal of As­(III) reached 76.3%, that of Cr­(VI) 100%, and that of F^–^ 22.2%. After 10 performance cycles, the nanocomposite shows decreases in their efficiency of only 26% for As­(III), 33% Cr­(VI), and 16% F^–^. A gradual decrease in regeneration ability was observed within each cycle, due to certain functional groups on the CS/kCar@GO surface not being fully regenerated or due to the fact that small active molecules dissolved in the elution solution.^95^ Overall, the CS/kCar@GO material can be successfully reused multiple times for extraction of the studied pollutants.

Figure 12 illustrates the adsorption capacity of CS/kCar@GO for As­(III), Cr­(VI), and fluoride ions during the regeneration study. Specifically, in the first regeneration cycle, the composite removed 0.15 mg of As­(III) per gram, 1.0 mg of Cr­(VI) per gram, and 2.22 mg of fluoride ions per gram. As the regeneration cycles progressed, the material’s ability to remove pollutants gradually declined. This reduction in performance is likely attributed either to the retention of pollutants within the internal structure of the composite or to structural degradation of the material due to repeated use, ultimately leading to a decrease in its adsorption capacity.

**Figure 12:**
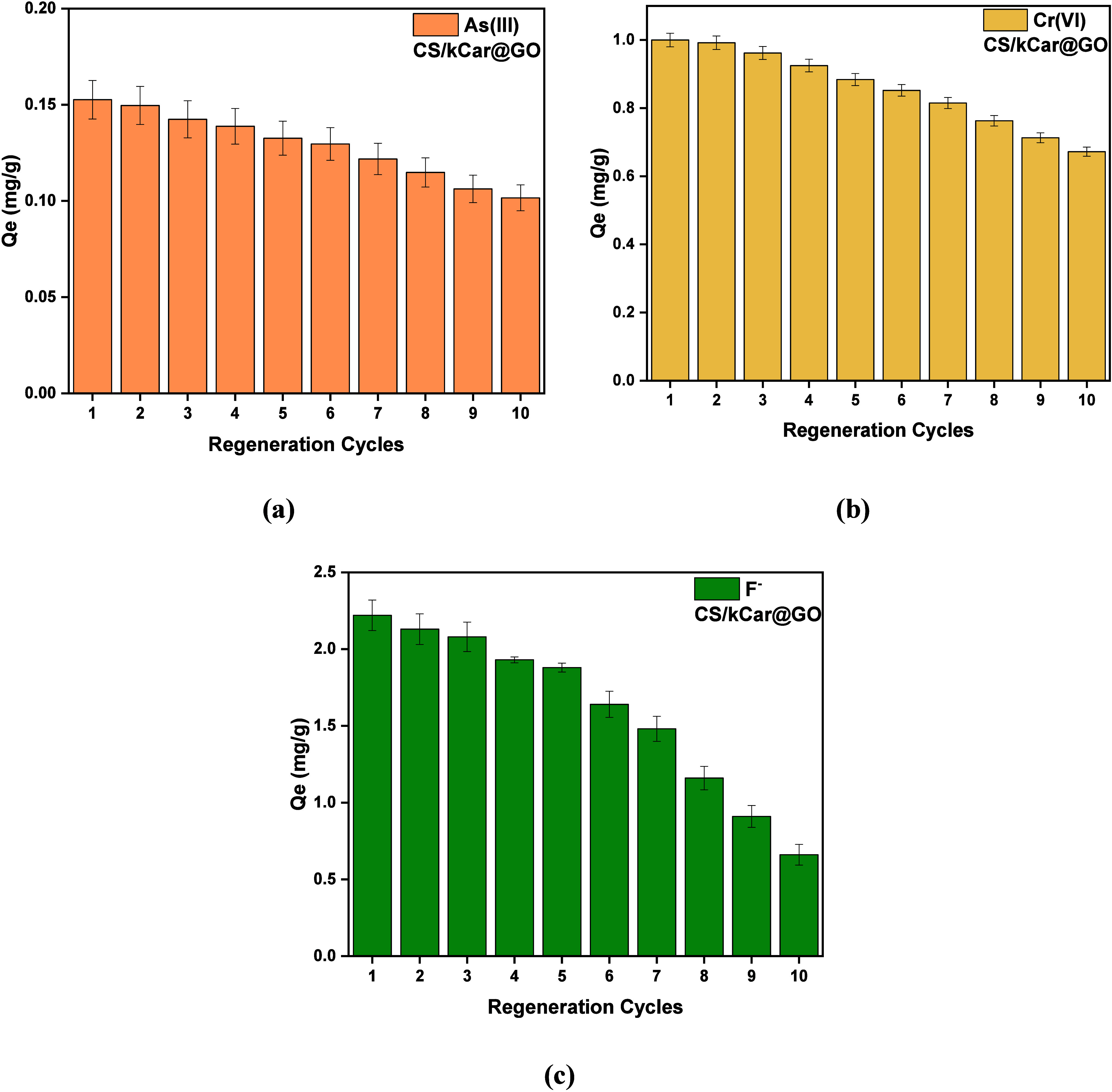
Adsorption capacity of (a) As­(III), (b) Cr­(VI), and (c) fluoride ions during the regeneration study: 0.1 mg/L As­(III) at pH 5.0, 0.5 mg/L Cr­(VI) at pH 7.0, and 5 mg/L F^–^ at pH 3.0 for 2 h.

Successful regeneration removes heavy metals and fluoride ions without significantly affecting the high adsorption efficiency of CS/kCar@GO. This makes CS/kCar@GO a viable and economical solution for wastewater treatment. In summary, CS/kCar@GO combines efficiency, chemical and mechanical strength, and easy regeneration; for this reason, it is a promising solution for the removal of heavy metals and fluoride ions from wastewater.

### Stability Study

In Figure 13, the results of the stability studies of optimum CS/kCar/GO at different pH values (3.0, 5.0, and 7.0) are presented. It is observed that CS/kCar/GO exhibits greater stability as an adsorbent material, particularly at pH 5.0. This suggests that the incorporation of GO enhances the stability and strengthens the compactness of the chemical structure. It is known that GO has many functional groups on its surface, which enhance its interaction with other materials through strong intermolecular bonds. In particular, GO can contribute to increasing the surface area overall stability of CS/kCar@GO.^96^ Additionally, GO helps minimize the degradation of the adsorbent material, enhancing its mechanical and chemical properties, which is crucial for environmental applications.^97^ Li et al. studied the adsorption of lysozyme using CS, sodium alginate (SA), and GO. The results showed that the CS/SA/GO adsorbent material exhibited enhanced mechanical, structural, and thermal stability due to the presence of GO. Subsequently, the addition of GO to the polymer matrix significantly improved the overall stability of the material, making it more robust and durable.^98^

**Figure 13:**
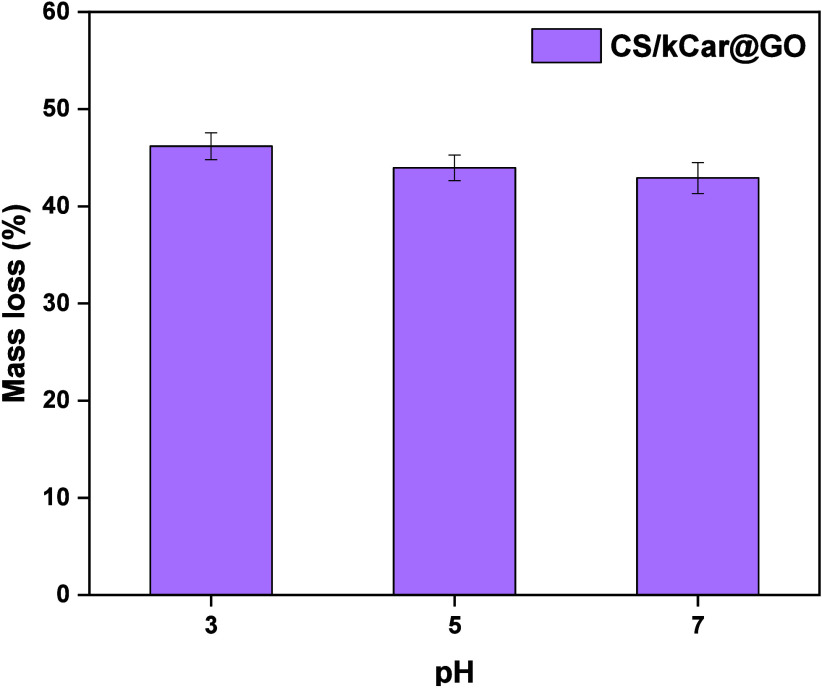
Stability study of kCar, CS/kCar, and CS/kCar/GO at different pH values.

### Comparison with the Literature

The adsorbent material CS/kCar@GO seems to be an excellent choice for the removal of heavy metals and fluoride ions from wastewater because it shows excellent adsorbent results in all three categories of pollutants. In the case of As­(III), other adsorbent materials such as Ce-CNB,^99^ CMGO,^3^ and MGOCS^100^ have higher removal efficiency or higher adsorption capacity in specific cases, but CS/kCar@GO stands out for its balance between performance and flexibility. Specifically, the removal efficiency of Cr­(VI) was 100%, which means that CS/kCar@GO can completely remove Cr­(VI) from wastewater. Even though the maximum adsorption capacity (*Q*_m_ = 2.82 mg/g) is relatively low, the total removal efficiency makes CS/kCar@GO a suitable adsorbent material for removing Cr­(VI) at medium concentrations. Finally, in the case of fluoride ions, CS/kCar@GO demonstrates an excellent equilibrium between the adsorption efficiency and adsorption capacity. This high capacity makes CS/kCar@GO more efficient than other adsorbent materials (Table 6) that may exhibit higher removal efficiency but have very low adsorption capacity.

**Table 6:** Comparison of CS/kCar@GO with Other Contiguous Adsorbents Found in the Literature for As­(III), Cr­(VI), and F^–^ Removal

adsorbent	*C*_0_ (mg/L)	dosage (g/L)	pH	removal (%)	*Q*_m_ (mg/g)	ref
As(III)						
Ce-CNB	30	0.2	8	18	57.5	^99^
CMGO	50	5	7	61	45	^3^
MGOCS	5	1	6	5.81	0.25	^101^
CS/kCar@GO	0.1	0.1	5	79	2.44	this study
Cr(VI)						
rGO-ZnO	50	0.01	3	68	25.45	^102^
CS-GO	50	2	2	96	104.16	^103^
Fe_2_O_3_-GO/CS	80	0.9	2	94.3	79.6	^104^
CS/kCar@GO	0.5	0.5	7	100	2.82	this study
F^–^						
AC-Mg	10	0.5	7	92	36.56	^73^
calcium phosphate-chitosan adsorbents	9.6	5	5.2	45	132.25	^105^
carboxylated chitosan magnetic	5	2	3	91	0.288	^106^
CS/kCar@GO	5	0.5	3	35.8	32.63	this study

In summary, CS/kCar@GO proves to be an optimum choice due to its wide use and its ability to remove various pollutants. Its excellent performance in the removal of Cr­(VI), as well as its very satisfactory application for As­(III) and F^–^, makes it a useful material for wastewater treatment.

## Conclusions

In this work, CS/kCar and CS/kCar@GO nanocomposites were developed as adsorbents, from natural biopolymers such as chitosan and kappa carrageenan, which were chemically cross-linked using GLA, PVA, and GO. Formed nanocomposites were characterized using FTIR, XRD, and SEM. The characterization results revealed that the addition of GO enhances the physicochemical properties of the nanocomposite. The SEM images confirmed that the incorporation of GO into the polymeric matrix results in a denser structure with a smoother surface. XRD patterns indicated the amorphous nanocomposites’ form. FTIR results after adsorption revealed that the studied pollutants interact with the functional group of the nanocomposites; thus, the kinetics, thermodynamics, and hydrogen bonding play a key role in the adsorption of As­(III), Cr­(VI), and F^–^ by CS/kCar@GO.

The removal process revealed that pH 5.0 for As­(III), pH 7.0 for Cr­(VI), and pH 3.0 for F^–^, at 293 K, were the optimum values when 0.5 g/L adsorbent was added for all experiments. CS/kCar@GO demonstrated significant potential for wastewater treatment, achieving removal efficiencies of 79.3% for As­(III), 100% for Cr­(VI), and 35.8% for F^–^. Adsorption followed a pseudo-second-order kinetic model, and the Langmuir isotherm provided the best fit for the data. The maximum adsorption capacities were found to be 2.44 mg/g for As­(III), 2.82 mg/g for Cr­(VI), and 32.63 mg/g for F^–^. Thermodynamic analysis indicated that the adsorption process was spontaneous for As­(III), Cr­(VI), and F^–^. Desorption of CS/kCar@GO was achieved by using 0.01 M NaOH, and the material remained effective for up to 10 cycles of reuse. According to swelling studies, it was assumed that the swelling is a two-stage process; the first is fast, and the second is much slower. After 60 min, the swelling reaches a plateau.

## Supplementary Material



## Data Availability

Data will be made available upon request.

## Notes

FTIR spectra of the pure materials kCar and CS (Figure S1) (PDF)
