# Supplementary material for: Carrageenan@Graphene Oxide Nanocomposites for the Adsorption of Trivalent Arsenic, Hexavalent Chromium, and Fluoride Ions from Aqueous Solutions: Conceptualization Modeling of Adsorptive Interfacial Interactions, Kinetics, and Swelling
Source: Langmuir. 2025 Jun 23;41(25):16529–47. doi: 10.1021/acs.langmuir.5c01927 (PMC12224336; doi:10.1021/acs.langmuir.5c01927)
Supplement: Supplementary file 1 [file la5c01927_si_001.pdf]

Carrageenan@graphene oxide nanocomposites for the adsorption of trivalent arsenic, hexavalent chromium and fluoride ions from aqueous solutions: Conceptualization modelling of adsorptive interfacial interactions, kinetics, and swelling

Anastasia D. Meretoudi[1], Athanasia K. Tolkou[1], Ramonna I. Kosheleva[1], Maria Xanthopoulou[2], Nikolaos M. Tzollas[2], Margaritis Kostoglou[2], Ioannis A. Katsoyiannis[2], George Z. Kyzas[1,*]

[1] *Hephaestus Laboratory, School of Chemistry, Faculty of Sciences, Democritus University of Thrace, GR-65404 Kavala, Greece (ameretou@chem.duth.gr (A.D.M.); atolkou@chem.duth.gr (A.K.T.); rkosheleva@chem.duth.gr (R.I.K); kyzas@chem.duth.gr (G.Z.K.))*
[2] *Laboratory of Chemical and Environmental Technology, Department of Chemistry, Aristotle University of Thessaloniki, GR-54124, Thessaloniki, Greece (mariaxanth@chem.auth.gr (M.X.); ntzollas@gmail.com (N.M.T); kostoglu@chem.auth.gr (M.K.); katsogia@chem.auth.gr (I.A.K.))*

*Corresponding author: kyzas@chem.duth.gr (G.Z.K.), *Hephaestus Laboratory, School of Chemistry, Faculty of Sciences, Democritus University of Thrace, GR 65404 Kavala, Greece, Tel.: +30-2510-46-2218*

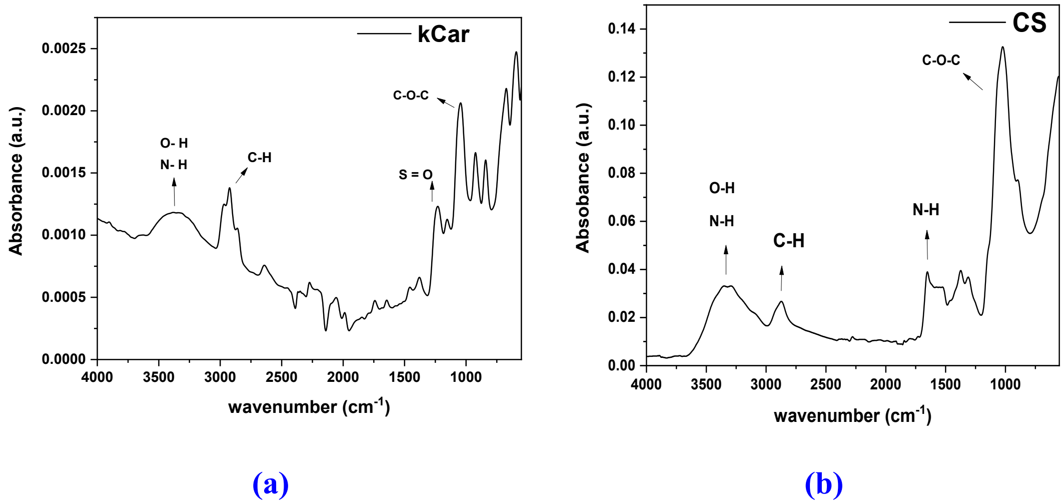

Figure S1. FT-IR spectra of pure kCar and CS.
